# No association of malignant B‐cell non‐Hodgkin lymphomas with ipsilateral SARS‐CoV‐2 vaccination

**DOI:** 10.1002/cam4.5687

**Published:** 2023-02-12

**Authors:** Luise Victoria Claaß, Patrick Mayr, Lisa Paschold, Thomas Weber, Denis Terziev, Bertram Jehs, Richard Brill, Johannes Dober, Bruno Märkl, Claudia Wickenhauser, Piotr Czapiewski, Martin Trepel, Rainer Claus, Mascha Binder

**Affiliations:** ^1^ Department of Internal Medicine IV, Oncology/Hematology Martin‐Luther‐University Halle‐Wittenberg Halle (Saale) Germany; ^2^ Department of Hematology and Oncology, Medical Faculty University of Augsburg Augsburg Germany; ^3^ Department of Diagnostic and Interventional Radiology, Medical Faculty University of Augsburg Augsburg Germany; ^4^ Clinic and Policlinic of Radiology Martin‐Luther University Halle‐Wittenberg Halle (Saale) Germany; ^5^ General Pathology and Molecular Diagnostics, Medical Faculty University of Augsburg Augsburg Germany; ^6^ Institute of Pathology, University Hospital Halle (Saale), Martin‐Luther‐University Halle‐Wittenberg Halle Germany; ^7^ Department of Pathology, Medical Faculty Otto‐Von‐Guericke University Magdeburg Magdeburg Germany; ^8^ Department of Pathology Dessau Medical Centre Institute of Pathology Dessau Germany; ^9^ Comprehensive Cancer Center Augsburg (CCCA), Medical Faculty University of Augsburg Augsburg Germany

**Keywords:** B‐cell non‐Hodgkin lymphoma, malignant B‐cell receptor, SARS‐CoV‐2 vaccination, unilateral lymphoma site, VDJ rearrangement

## Abstract

**Purpose:**

SARS‐CoV‐2 vaccines cause acute ipsilateral lymph node swelling in an important proportion of vaccines. Thus far, no malignant lymphadenopathies have been reported in temporal context to vaccination in the ipsilateral draining lymph node areas.

**Experimental design:**

Prompted by two cases with unilateral axillary lymphomas that occurred ipsilaterally to prior SARS‐CoV‐2 vaccination, we systematically retrieved all B‐cell non‐Hodgkin lymphomas at two German University Medical Centers diagnosed before and after introduction of SARS‐CoV‐2 vaccines in Germany. Available lymphoma tissue (n=19) was subjected to next‐generation immunosequencing of the IGH locus. Malignant clonotypes were mined in the CoVabDab database and published data sets from 342 uninfected individuals, 55 individuals 28 days after anti‐SARS‐CoV‐2 vaccination and 139 individuals with acute COVID‐19 together encompassing over 1 million CDR3 sequences in total.

**Results:**

Of 313 newly diagnosed cases in the two centers and observation periods, 27 unilateral manifestations in the defined deltoid draining regions were identified. The majority thereof were diffuse large B‐cell lymphomas (18 of 27 cases). Eleven unilateral cases were diagnosed in the era of SARS‐CoV‐2 vaccination and 16 in the control period before introduction of such vaccines. Of the 11 unilateral lymphomas that occurred during the vaccination period, ten had received a SARS‐CoV‐2 vaccine prior to lymphoma diagnosis. These cases were further evaluated. While left‐sided were more frequent than right‐sided lymphomas (19 vs 8 cases), no statistically significant association of vaccination site and laterality of the lymphoma manifestation was found. The unilateral lymphomas showed a normal range of B‐cell receptors typically found in these lymphoma subtypes with no evidence for anti‐SARS‐CoV‐2 sequences in the malignant clonotype.

**Conclusions:**

Together, we found no evidence that the current SARS‐CoV‐2 vaccines could serve as a trigger for lymphomagenesis in the draining lymph node areas of the deltoid region used for vaccination.

## Graphical abstract

Systematical screening including NGS immunosequencing of B‐cell non‐Hodkin lymphomas at two German University Meidcal Ceners diagnosed before and after introduction of SARS‐CoV‐2 vaccines. There was no evidence that the current SARS‐CoV‐2 vaccines could serve as a trigger for lymphomagenesis.

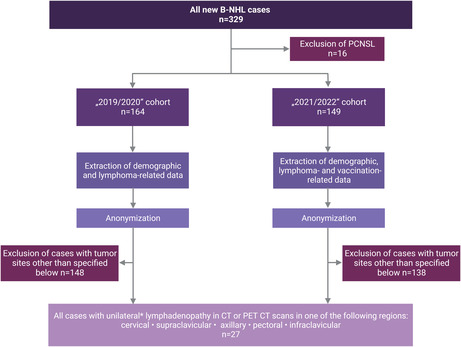

## 1 INTRODUCTION

Vaccination‐associated lymphadenopathy is a well‐known phenomenon frequently observed upon application of different vaccines. With the advent of worldwide SARS‐CoV‐2 immunizations, vaccination‐associated benign ipsilateral axillary and supraclavicular lymphadenopathies have been reported.^1^, ^2^, ^3^ the observed lymphadenopathies are well compatible with the recommended injection site of SARS‐CoV‐2 vaccines, which is the deltoid region draining into axillary and supraclavicular lymph node areas.^2^, ^4^ in some healthy individuals, major lymph node enlargement has prompted investigation for suspected malignancy.^2^ Some studies suggested that axillary swelling and tenderness caused by the immunological reaction to the vaccine were more common with the Moderna SARS‐CoV‐2 vaccine (up to 16% versus <2% with the BioNTech vaccine).^5^, ^6^ While clinically detectable lymphadenopathy by physical examination remains a rare adverse event (5% of all lymphadenopathies in a literature review with a total of 2057 patients included), a number of imaging studies including positron emission tomography (PET)/computed tomography (CT), magnetic resonance imaging, and ultrasound reported an incidence of postvaccination lymphadenopathy between 14.5% (after a single dose) and 53% (after >1 doses).^4^ These case series alerted oncologists to interpret unexpected axillary or supraclavicular signals on such scans with caution and always in the context of prior SARS‐CoV‐2 vaccination.^2^, ^7^

In 2021, we newly diagnosed two lymphoma cases involving almost exclusively the axillary lymph nodes that occurred after ipsilateral SARS‐CoV‐2 immunization. This prompted us to systematically analyze unilateral lymphomas arising in lymph nodes within the potential draining area of SARS‐CoV‐2 vaccines in two independent University Medical Centers in Germany. This analysis yielded no evidence for lymphoma association with prior ipsilateral SARS‐CoV‐2 vaccination further supporting the safety of these vaccines.

## 2 METHODS

### 2.1 Cohorts

**FIGURE 1:**
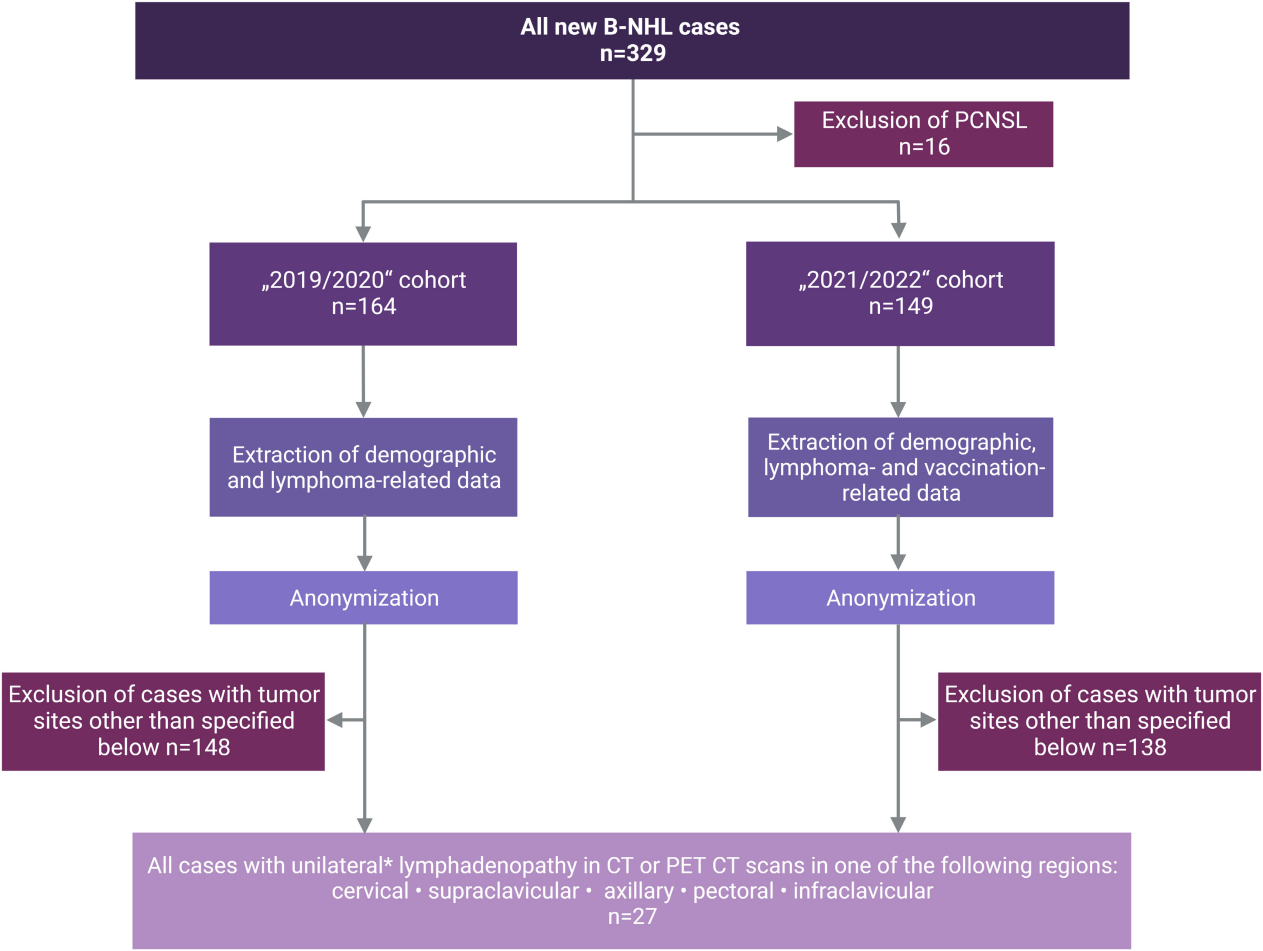
Schematic study overview. A list of all B‐cell non‐Hodgkin lymphoma (B‐NHL) cases (apart from primary central nervous system lymphomas, PCNSL) newly diagnosed in the era after the introduction of SARS‐CoV‐2 vaccines (01/01/2021–31/03/2022; 2021/2022 cases) as well as in a control period prior to introduction of SARS‐CoV‐2 vaccines (01/10/2019–12/31/2020; 2019/2020 cases) was extracted from electronical records. Consecutively, information on lymphoma subtype, age, sex, pattern of involved sites, CT/PET CT scans (reports and images), and vaccination history was retrieved from electronical records. All cases with unilateral manifestations in regions that represent the draining area of the deltoid vaccine injection site were included. This analysis was performed by a blinded radiologist. Patients with bilateral lymphadenopathy were excluded.

Two cohorts were retrospectively assembled at two University Medical Centers (Halle and Augsburg) according to the same algorithm (schematically shown in Figure 1).^8^, ^9^, ^10^, ^11^ In brief, defined information on all B‐cell non‐Hodgkin lymphoma (B‐NHL) cases newly diagnosed in the era after the introduction of SARS‐CoV‐2 vaccines (01/01/2021–31/03/2022; 2021/2022 cases) as well as in a control period prior to introduction of SARS‐CoV‐2 vaccines (01/10/2019–12/31/2020; 2019/2020 cases) was retrieved from electronical records. This included information on lymphoma subtype, age, sex, pattern of involved sites, CT/PET CT scans (reports and images), and vaccination history. All cases with unilateral manifestations in the following regions that represent the potential draining area of the deltoid vaccine injection site were included: cervical, supraclavicular, axillary, pectoral, and infraclavicular. This analysis was performed by a blinded radiologist. Patients with bilateral lymphadenopathy and unilateral manifestation others than specified were excluded. The only excluded type of B‐cell non‐Hodgkin lymphoma was primary central nervous system lymphoma (PCNSL), since this type of lymphoma—by definition—cannot be located in the draining region of SARS‐CoV‐2 vaccines. Of all unilateral cases meeting the inclusion criteria, paraffin‐embedded tissue from diagnostic samples was retrieved from the pathology archive and all cases were subsequently anonymized.

### 2.2 Next‐generation sequencing of the immunoglobulin heavy chain (IGH) locus to determine the malignant lymphoma's B‐cell receptor clonotype

Amplification of V‐D‐J rearrangements from the *IGH* locus was done as described elsewhere on genomic DNA extracted from diagnostic lymphoma tissue.^12^ Sequencing and demultiplexing were performed on the Illumina MiSeq platform with 2 × 301 cycles at a coverage of 80,000 reads per sample. Analysis of the immunoglobulin heavy chain (IGH) locus was computed using the MiXCR analysis tool V.3.0.12.

We retrieved a set of 2778 SARS‐CoV‐2 binding human BCR sequences from CoVAbDab^13^ (accessed 2nd March 2022) and searched for exact matches or similar sequences up to Levenshtein distance 2 with the malignant CDR3 amino acid sequences of the unilateral lymphoma cases from Halle and Augsburg (*n* = 5). As there was no overlap, we next mined previously published IGH repertoires of 342 uninfected, unvaccinated healthy donors (HD) covering 678,620 unique CDR3 sequences, 55 individuals within 28 days after anti‐SARS‐CoV‐2 vaccination covering 149,862 unique CDR3 sequences^14^ and 139 individuals at acute COVID‐19 infection covering 354,971 unique CDR3 sequences.^15^, ^16^ We searched these repertoires for exact matches of the CDR3 amino acid sequence with the unilateral lymphoma cases (n = 5) and with the SARS‐CoV‐2 binding human sequences from CoVAbDab (*n* = 2778). All analyses were performed using RStudio and R version 4.2.0. and packages vwr and ggplot2.^17^

## 3 RESULTS

### 3.1 Description of index cases HC1 and HC2

During the year 2021, when SARS‐CoV‐2 vaccines first became available in Germany, two cases with left‐sided axillary lymphadenopathy presented at the University Medical Center Halle (Saale). The lymphadenopathy was histologically confirmed as diffuse large B‐cell lymphoma (DLBCL) in both patients. The respective index patients reported prior SARS‐CoV‐2 vaccination at the left arm raising our suspicion that the lymphoma could have been promoted or even induced by the vaccination.

### 3.2 Lymphoma cases with unilateral lymph node involvement diagnosed in 2021/2022 versus a control period in 2019/2020

To systematically analyze a potential association of lymphomas in the draining region of SARS‐CoV‐2 vaccines with prior ipsilateral vaccination, we extracted data from all consecutive newly diagnosed B‐NHL patients at the University Medical Center Halle (Halle cohort) and—as a second independent cohort—at the University Medical Center Augsburg (Augsburg cohort). The 2021/2022 cases comprised lymphoma patients that had been diagnosed after licensing of SARS‐CoV‐2 vaccines in Germany. In the Halle cohort, overall 65 B‐NHL cases (excluding PCNSL) were diagnosed in this period. Of these, five cases were unilaterally located in the potential draining region of SARS‐CoV‐2 vaccines as defined by our algorithm (Figure 1). Four of them were left‐sided and one case right‐sided. The four left‐sided lymphomas included the two index patients that had triggered this analysis. All of the five lymphoma cases were DLBCL, three of germinal‐center B‐cell (GCB) subtype and two of non‐GCB subtype. In the Augsburg cohort, overall 84 B‐NHL cases (excluding PCNSL) were diagnosed in this period. Of these, six unilateral cases were diagnosed in 2021/2022 that fulfilled the criteria defined by our algorithm. Four cases were left‐sided and two cases right‐sided. These included three follicular lymphomas (FL), two DLBCL (one non‐GCB subtype, the other undefined) and one mantle cell lymphoma (MCL). Of these cases, the left‐sided mantle cell lymphoma had not received a SARS‐CoV‐2 vaccination prior to lymphoma diagnosis and was therefore excluded from the vaccination cohort‐specific analysis.

As a historical control, all unilateral lymphomas cases in the defined regions were retrieved at the two centers in a corresponding time period before introduction of SARS‐CoV‐2 vaccines. In the Halle 2019/2020 control cohort, a total of 72 cases were diagnosed, thereof five unilateral cases with three left‐sided and two right‐sided lymphomas. Histologically, these were four DLBCL, all non‐GCB subtype, and one FL. In the Augsburg 2019/2020 control cohort, of 92 total cases, 11 lymphomas were unilateral, including eight left‐sided and three right‐sided cases. Histologic subtypes comprised seven DLBCL and four FL.

**FIGURE 2:**
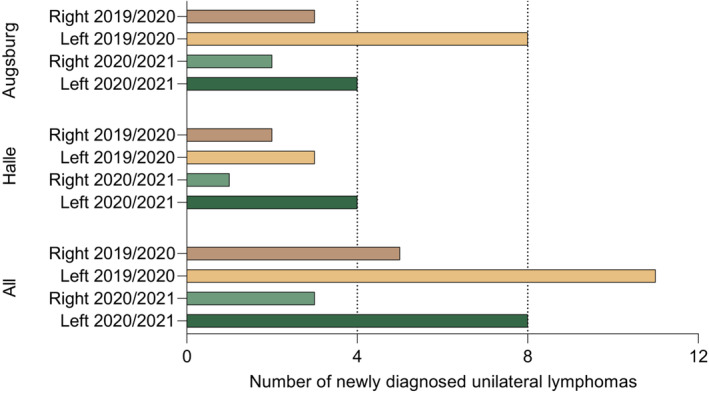
Sidedness of newly diagnosed B‐NHL in the 2019/2020 and 2021/2022 cohorts.

Taking together, 27 unilateral lymphomas in the defined locations were retrieved from the records of both medical centers making up a frequency of 8,6% of all diagnosed lymphomas. This frequency of unilateral lymphomas in the defined regions was similar before (9.8%) and after (7.4%) the introduction of SARS‐CoV‐2 vaccines. Interestingly, with 19 left‐sided out of 27 unilateral cases, these were more frequent than right‐sided cases. The numbers of left‐ and right‐sided cases were significantly different across all four subgroups (Halle and Augsburg cohorts, both observation periods; *T*‐test *p* = 0.024). Detailed information on all unilateral cases from both cohorts is summarized in Table 1. Figure 2 illustrates the sidedness of all cases.

**TABLE 1:** Unilateral lymphoma cases in the deltoid draining lymph node regions.

Case number	Cohort	Sex	Age at diagnosis (decade)	Lymphoma subtype	Involved side
HC1	Halle 2019/2020	Female	81–90	DLBCL	Right
HC2	Halle 2019/2020	Female	61–70	DLBCL	Left
HC3	Halle 2019/2020	Female	71–80	DLBCL	Left
HC4	Halle 2019/2020	Male	21–30	DLBCL	Left
HC5	Halle 2019/2020	Female	61–70	FL	Right
HC6	Halle 2021/2022	Male	51–60	DLBCL	Left
HC7	Halle 2021/2022	Male	61–70	DLBCL	Left
HC8	Halle 2021/2022	Female	71–80	DLBCL	Left
HC9	Halle 2021/2022	Female	71–80	DLBCL	Left
HC10	Halle 2021/2022	Male	71–80	DLBCL	Right
AC1	Augsburg 2019/2020	Female	61–70	FL	Left
AC2	Augsburg 2019/2020	Male	31–40	DLBCL	Left
AC3	Augsburg 2019/2020	Male	51–60	DLBCL	Left
AC4	Augsburg 2019/2020	Female	71–80	FL	Left
AC5	Augsburg 2019/2020	Male	81–90	DLBCL	Left
AC6	Augsburg 2019/2020	Male	61–70	DLBCL	Left
AC7	Augsburg 2019/2020	Female	61–70	FL	Right
AC8	Augsburg 2019/2020	Female	71–80	FL	Left
AC9	Augsburg 2019/2020	Male	71–80	DLBCL	Right
AC10	Augsburg 2019/2020	Female	71–80	DLBCL	Left
AC11	Augsburg 2019/2020	Female	71–80	DLBCL	Right
AC12	Augsburg 2021/2022	Male	N/A	MCL	Left
AC13	Augsburg 2021/2022	Male	71–80	FL	Left
AC14	Augsburg 2021/2022	Male	71–80	DLBCL	Right
AC15	Augsburg 2021/2022	Male	61–70	FL	Left
AC16	Augsburg 2021/2022	Female	61–70	DLBCL	Right
AC17	Augsburg 2021/2022	Female	91–100	FL	Left

### 3.3 Vaccination history

Ten of the 11 unilateral lymphoma cases in the Halle and Augsburg 2021/2022 cohort had been vaccinated with SARS‐CoV‐2 vaccines prior to lymphoma diagnosis.

**FIGURE 3:**
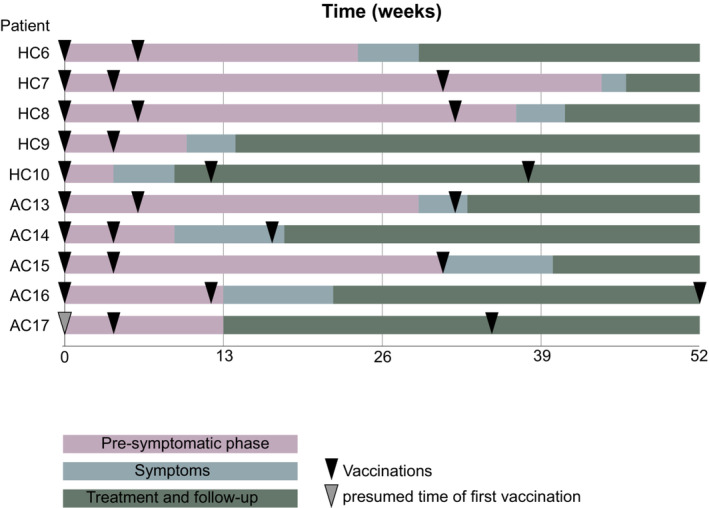
Individual disease courses and timing of vaccination in patients with newly diagnosed B‐NHL in the 2021/2022 cohort. In Patient AC17, the date of the first vaccination could not be determined. We included an approximated time of vaccination four weeks prior to the documented second vaccination.

All four patients with the left‐sided lymphomas from the Halle 2021/2022 cohort had received a dose of Moderna as their last vaccine in the left deltoid region. This last vaccination had been received 2–5 months prior to lymphoma diagnosis as shown in Figure 3. The patient with right‐sided lymphoma from the Halle 2021/2022 cohort had received one dose of AstraZeneca in the left deltoid region prior to lymphoma diagnosis.

In the Augsburg 2021/2022 cohort, all four patients with vaccinations prior to lymphoma diagnosis received the BionTech vaccine with one patient additionally receiving the Moderna vaccine. In one case, the type of vaccine could not be clarified. Three patients were vaccinated in the left deltoid region, two in the right deltoid region.

Taking together data from both medical centers, 5 out of 11 patients showed concordance of side of vaccination and lymphoma. The contingency table reflects the predominant left‐sidedness of the lymphomas and the preferential use of the left deltoid region for vaccination, as most people are right‐handed (Table 2). However, no statistically significant association (*p* = 1.0000) by Fisher's exact test was found between ipsilateral vaccination and lymphoma.

**TABLE 2:** Contingency table of lymphoma sidedness and site of prior SARS‐CoV‐2 vaccination.^a^

	SARS‐CoV‐2 vaccination	
	Left deltoid region	Right deltoid region
B‐NHL		
Left‐sided (deltoid draining region)	5	2
Right‐sided (deltoid draining region)	3	0

^a^ Sidedness of lymphoma and vaccination were independent of each other as assessed by Fisher's exact test.

### 3.4 Molecular profiling of unilateral lymphomas in the deltoid draining region

**FIGURE 4:**
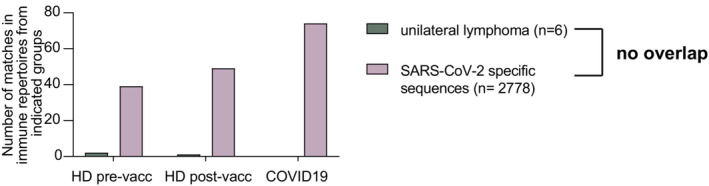
Mining B‐cell receptor repertoires of healthy, SARS‐CoV‐2 vaccinated or COVID‐19‐infected individuals for clonotypes of unilateral postvaccination lymphomas (*n* = 6) und anti‐SARS‐CoV‐2 clonotypes (*n* = 2778). Number of exact matches in CDR3 amino acid sequence with sequences from unvaccinated, uninfected healthy donors (HD pre‐vacc, *n* = 678,620), postvaccinated healthy donors (HD post‐vacc, *n* = 149,862) or individuals with active COVID‐19 infection (COVID‐19, *n* = 354,971).

All lymphomas with unilaterality from both cohorts were subjected to IGH sequencing to determine the respective V‐D‐J rearrangement of the malignant clone's B‐cell receptor. Of 19 available samples, 13 revealed an unambiguous IGH sequence reflecting the known sensitivity of the method for detecting the malignant clonotype in B‐NHL. These clonotypes showed a typical distribution of IGHV gene usage as previously published for these types of lymphoma entities^18^ (Table 3). Prior studies have generated extensive knowledge on the B‐cell receptor sequences able to recognize SARS‐CoV‐2 spike protein epitopes (e.g.^13^, ^15^). We reasoned that if vaccines encoding the spike protein of SARS‐CoV‐2 triggered lymphomas in the draining lymph node areas of the deltoid region, the B‐cell receptor of these lymphomas may belong to a class with known SARS‐CoV‐2 specificity. To test this, we aligned the sequences of the malignant lymphoma clonotypes with *n* = 2778 unique SARS‐CoV‐2 binding human CDR3 sequences retrieved from CoV‐AbDab.^13^ This analysis did not show evidence for typical anti‐SARS‐CoV‐2 B‐cell receptor sequences in the lymphoma cases with unilateral presentation. Next, we screened B‐cell receptor repertoires from healthy donors prevaccination and postvaccination and from individuals with an acute COVID‐19 infection for the presence of the unilateral lymphoma sequences. While the set of SARS‐CoV‐2‐specific sequences derived from CoVAbDab showed a clear enrichment in postvaccinated and COVID‐19‐infected individuals, no such pattern was observed for unilateral lymphoma sequences (Figure 4).

**TABLE 3:** V‐D‐J gene rearrangements of the malignant lymphoma clonotypes of unilateral cases. The mutation status reflects the number of mutations within the IGHV nucleotide sequence from the closest germline sequence. Unmutated (UM): more than 98% similarity, mutated (M): less than 98% similarity. Read depths information is included in Table S1.

Case number	Cohort	CDR3 amino acid sequence	IGHV	IGHD	IGHJ	Mutation status
HC1	Halle 2019/2020	CARGFGRGGYILTGYYYW	IGHV4/OR15‐8	IGHD3‐9	IGHJ4	M
HC2		No dominant clone identified	NA	NA	NA	NA
HC3		CARENADTGRALDSW	IGHV4‐31	IGHD6‐6	IGHJ4	M
HC4		No dominant clone identified	NA	NA	NA	NA
HC5		CARHWETLQE_TNCLHYFDYW	IGHV1‐8	IGHD2‐8	IGHJ4	UM
HC6	Halle 2021/2022	CARHSRSSTGPSDPFDPW	IGHV5‐51	IGHD2‐15	IGHJ5	UM
HC7		CTRNFYDSSGYFSWGGTFDYW	IGHV3‐71	IGHD3‐22	IGHJ4	M
HC8		CTRETHQFGSGSSDVW	IGHV3‐7	IGHD3‐10	IGHJ6	M
HC9		CARVLSTTAVRDTETHITSFYYGVDVW	IGHV4/OR15‐8	IGHD6‐6	IGHJ6	M
HC10		No dominant clone identified	NA	NA	NA	NA
AC1	Augsburg 2019/2020	No dominant clone identified	NA	NA	NA	NA
AC2		No material available	NA	NA	NA	NA
AC3		No dominant clone identified	NA	NA	NA	NA
AC4		CTQSTGTFRFDPW	IGHV3‐23D	IGHD2‐8	IGHJ5	UM
AC5		No material available	NA	NA	NA	NA
AC6		CVKEYPIEGLAGYW	IGHV3‐23D	IGHD3/OR15‐3a	IGHJ4	M
AC7		CARDKGGIFFGQLLPREGYFDCW	IGHV4‐30‐4	IGHD3‐3	IGHJ4	UM
AC8		CARHYTNYANWFDPW	IGHV3‐23D	IGHD2‐8	IGHJ5	UM
AC9		No material available	NA	NA	NA	NA
AC10		CARGQLSESYCFDYW	IGHV4/OR15‐8	IGHD2‐2	IGHJ4	UM
AC11		No material available	NA	NA	NA	NA
AC12	Augsburg 2021/2022	CAGTVNYDDYSGNYAMDVW	IGHV1‐69‐2	IGHD4‐17	IGHJ6	UM
AC13		CARDPDHADYPFFDYW	IGHV3‐7	IGHD2‐2	IGHJ4	UM
AC14		No material available	NA	NA	NA	NA
AC15		No dominant clone idenitified	NA	NA	NA	NA
AC16		No material available	NA	NA	NA	NA
AC17		No material available	NA	NA	NA	NA

## 4 DISCUSSION

The need to quickly immunize the world population against SARS‐CoV‐2 has resulted in emergency authorization of a considerable number of vaccines at unprecedented speed. Evidence of safety as an essential part of a vaccine‘s regulatory approval is gathered during all phases of the vaccine development process. In case of SARS‐CoV‐2 vaccines, some of the respective trials have lasted only very few months before regulatory approval thereby limiting the reporting of adverse events. This calls for caution and diligent reporting of potential adverse events after licensing by healthcare professionals.

While it has been clear from all licensing studies that SARS‐CoV‐2 vaccines may cause local pain, swelling, fever, and temporary malaise in a large proportion of patients,^14^, ^19^, ^20^, ^21^, ^22^, ^23^ a few rarer side effects have been increasingly reported after licensing such as myocarditis, autoimmune cytopenias, vaccine‐induced thrombotic thrombocytopenia, Guillain–Barré syndrome, or inflammatory arthritis.^24^, ^25^, ^26^

Two cases of unilateral axillary lymphomas diagnosed after ipsilateral SARS‐CoV‐2 vaccination in 2021 prompted us to systematically investigate unilateral lymphomas in the deltoid draining regions after and prior to introduction of SARS‐CoV‐2 vaccines. We reasoned that if SARS‐CoV‐2 vaccines triggered lymphomas in ipsilateral draining lymph node regions, a higher incidence of such ipsilateral lymphoma presentations should be expected in the era after introduction of the SARS‐CoV‐2 vaccines as compared to the era prior to SARS‐CoV‐2 vaccination. Moreover, we aimed to explore whether such unilateral lymphomas were associated with the side of vaccination. The study on 313 lymphoma cases revealed a frequency of 8,6% of unilateral lymphomas in the deltoid draining region, 7.4% after and 9.8% before introduction of SARS‐CoV‐2 vaccines. No indication for association of the sidedness of vaccination and lymphoma was found. Together with the normal B‐cell receptor profiles of these lymphomas without evidence for SARS‐CoV‐2 directed sequences, these data did not provide any support for the notion that SARS‐CoV‐2 vaccines trigger or promote ipsilateral lymphoma development. This finding is consistent with previous data from clinical trials evaluating SARS‐CoV‐2 vaccines, in which no suspicious findings were reported that might point in this direction.^5^, ^6^ Moreover, it complements several case series evaluating acute postvaccination lymphadenopathies which uniformly showed benign alterations.^1^, ^2^, ^3^

While this represents the major finding of our study, we made the unexpected observation that left‐sided lymphomas in the deltoid draining region appear to be more frequent than right‐sided lymphomas. This was similar in both observation periods with 2.7 times more left‐sided cases in the era of SARS‐CoV‐2 vaccines and 2.2 times more left‐sided cases before introduction of such vaccines. To our knowledge, a bias for lymphoma on the left side has not been previously reported and, in our opinion, there is no evident reason explaining such a location bias. Future analyses of larger cohorts and inclusion of additional lymph node regions may, however, be necessary to confirm this finding.

Overall, a major limitation of our work is (i) the restriction to two University Medical Centers, (ii) the overall limited number of cases studied, and (iii) the lack of data from cancer registries. The second limitation is hard to address under the conditions of german cancer registration which suffers from significant reporting delays. Such analyses may, however, be performed at a later point in time, when all cases from 2019 to 2022 will have been completely registered.

Together, our analysis speaks in favor of the safety of SARS‐CoV‐2 vaccines. Even if our data do not suggest a relationship between SARS‐CoV‐2 vaccination and lymphomagenesis, future population‐based analyses are necessary to definitively rule out this and other potential long‐term risks of SARS‐CoV‐2 vaccines.

## AUTHOR CONTRIBUTIONS

**Luise Claaß:** Conceptualization (supporting); data curation (lead); formal analysis (equal); methodology (equal); project administration (lead); visualization (equal); writing – original draft (supporting). **Patrick Mayr:** Data curation (supporting); investigation (equal); methodology (lead); resources (equal). **Lisa Paschold:** Formal analysis (equal); methodology (equal); visualization (equal); writing – original draft (supporting); writing – review and editing (equal). **Thomas Weber:** Data curation (supporting); resources (equal). **Denis Terziev:** Data curation (supporting); resources (equal). **Bertram Jehs:** Formal analysis (equal); validation (equal). **Richard Brill:** Formal analysis (equal); validation (equal). **Johannes Dober:** Formal analysis (equal); validation (equal). **Bruno Märkl:** Resources (equal); writing – review and editing (supporting). **Claudia Wickenhauser:** Resources (equal). **Piotr Czapiewski:** Resources (equal). **Martin Trepel:** Resources (equal); writing – review and editing (supporting). **Rainer Claus:** Conceptualization (lead); methodology (equal); supervision (equal); writing – original draft (supporting); writing – review and editing (supporting). **Mascha Binder:** Conceptualization (lead); funding acquisition (lead); supervision (equal); writing – original draft (lead); writing – review and editing (equal).

## FUNDING INFORMATION

This project was funded by the Martin‐Luther‐University Halle (Saale).

## CONFLICT OF INTEREST STATEMENT

The authors declare no conflict of interests.

## ETHICS STATEMENT

This retrospective anonymized analysis of unilateral lymphoma cases was conducted according to the guidelines of the Declaration of Helsinki. It is in accordance with local general data protection regulation (§15 of the professional regulations of the Medical Association of Saxony‐Anhalt) and the Bavarian Hospital Act (“Bayerisches Krankenhausgesetz”). Written informed consent was received from participants who donated blood for NGS analysis prior to inclusion in the study. The study has been performed in accordance with the declaration of Helsinki of 1975.

## Supporting information


Table S 1:

## Data Availability

The NGS datasets generated for this study can be found in the European Nucleotide Archive (ENA) under accession number PRJEB54819.
